# Effect of pyrite on the treatment of chlorophenolic compounds with zero-valent iron-Fenton process under uncontrolled pH conditions: reaction mechanism and biodegradability

**DOI:** 10.1007/s11356-024-34329-z

**Published:** 2024-07-15

**Authors:** Ozlem Oral, Cetin Kantar, Ilker Yildiz

**Affiliations:** 1 Department of Environmental Engineering, Canakkale Onsekiz Mart University, 17100 Canakkale, Turkey; 2 Central Laboratory, Middle East Technical University, 06800 Ankara, Turkey

**Keywords:** Heterogeneous Fenton, Oxidative degradation, Wastewater treatment, Advanced oxidation processes, Adsorption, Enhanced biodegradability, 2,4-dichlorophenol, 4-chlorophenol

## Abstract

This current study explored the effect of pyrite on the treatment of chlorophenolic compounds (CP) by Fenton process with micron-sized zero-valent iron (ZVI) as the catalyst. The experiments were conducted in batch reactors with 100 mg L^−1^ CP, 0–0.02 M H_2_O_2_, and variable pyrite and ZVI doses (0–1 g L^−1^). Our findings show that while the reactor with 1 g L^−1^ ZVI as the only catalyst achieved only 10% CP removal efficiency due to rapid ZVI surface passivation and ZVI particle aggregation, the CP removal efficiency increased with increasing pyrite dose and reached 100% within couple of minutes in reactors with 0.8 g L^−1^ pyrite and 0.2 g L^−1^ ZVI. The CP removal was mainly driven by the oxidative treatment of CPs with some strong radicals such as hydroxyl radicals (•OH) while the adsorption onto the catalyst surface was only responsible for 10 to 25% of CP removals, depending on the type of CP studied. The positive impact of pyrite on CP removal by the ZVI/H_2_O_2_ system could be attributed to the ability of pyrite to (1) create an acidic environment for optimum Fenton process, (2) provide support material for ZVI to minimize ZVI particle agglomeration, and (3) stimulate iron redox cycling for improved surface site generation. Following oxidative Fenton treatment, the degradation intermediate products of CPs, including some aromatic compounds (benzoquinone, hydroquinone, etc.) and organic acids (e.g., acetic acid), became more biodegradable in comparison to their mother compounds. Overall, the treatment systems with a mixture of ZVI and pyrite as catalyst materials could offer a suitable cost-effective technology for the treatment of wastewater containing biologically non- or low-degradable toxic compounds such as chlorophenols.

**Supplementary Information:**

The online version contains supplementary material available at 10.1007/s11356-024-34329-z.

## Introduction

Chlorophenolic compounds (CPs) are widely used in several industrial applications, including pharmaceuticals and paint manufacturing (Liu et al. 2021; Yadav et al. 2023; Lv et al. 2024). Most CPs are highly toxic and pose a significant threat to the environment (Ezzatahmadi et al. 2019; Yadav et al. 2023). Due to their toxic nature, these compounds create some severe operational problems such as loss of biomass in biological wastewater treatment plants (Yadav et al. 2023). As an alternative to biodegradation, various treatment methods, including membrane technology, adsorption, and aeration, have been tested for the treatment of CPs (Garba et al. 2019; Yadav et al. 2023). Nevertheless, long reaction times, high cost and low removal efficiencies hinder their use in practical applications (Ezzatahmadi et al. 2019). Recently, different advanced oxidation processes have been, successfully, developed to eliminate or minimize the toxicity level of wastewaters containing toxic compounds prior to biodegradation (Ezzatahmadi et al. 2019; Yadav et al. 2023). Fenton process, for instance, offers a viable choice for toxicity reduction for chlorophenol-containing wastewaters (Liu et al. 2021; Kayan et al. 2021). However, the conventional Fenton process comes with some serious problems, including the necessity of highly acidic conditions for optimum Fenton operation, low iron recovery, high chemical cost, and excessive sludge generation (Kantar et al. 2019; Liu et al. 2021).

Recently, several researchers have proposed to use iron-containing materials such as zero-valent iron (ZVI) as the iron source in the Fenton process, owing to its cost-effectiveness, strong reactivity, and widespread accessibility (Guan et al. 2022; Liu et al. 2023; Yadav et al. 2023; Lv et al. 2024). The corrosion of ZVI by oxygen and hydrogen peroxide (H_2_O_2_) generates Fe^2+^ ions, which, then, interacts with H_2_O_2_ to produce hydroxyl radicals (•OH) in the heterogeneous Fenton process (Xu et al. 2019; Liu et al. 2021; Oral et al. 2024a). However, the use of ZVI as a catalyst in the Fenton reaction is limited because of the aggregation and surface passivation of ZVI particles, which reduces its reactivity and reusability (Ezzatahmadi et al. 2019; Guan et al. 2022). According to Bao et al. (2020), for instance, the aggregation of nano-ZVI particles due to strong dipole–dipole interaction significantly decreased the number of reactive sites on the ZVI surface. Recently, new surface modifications, including bimetallic particles, support materials, and surface sulfidation, have been investigated to overcome these shortcomings (e.g., Ezzatahmadi et al. 2019; Xu et al. 2019; Bao et al. 2020; Diao and Chu 2021; Sulaiman and Al-Jabari 2021; Sun et al. 2022; Wu et al. 2023). Most of these materials have been synthesized in the labs and successfully applied in the treatment of recalcitrant organic contaminants (e.g., Liu et al. 2021; Lv et al. 2024). However, the procedures for the synthesis of these synthetic materials may be too complex, and expensive. Due to its high S content, for instance, a naturally occurring low-cost mineral, pyrite, could serve as an S source for the surface sulfidation of ZVI, which facilitates the passage of electrons from the ZVI core to species that are attached to its surface (Du et al. 2019; Min et al. 2021). Pyrite can also act as a co-catalyst with ZVI in the Fenton process because of its high Fe content. Moreover, the process of pyrite oxidation by O_2_ and H_2_O_2_ results in the production of sulfate ions (SO_4_^2−^) and hydrogen ions (H^+^), which leads to a significant decline in solution pH to a level below 4, which is considered optimal for Fenton reactions (Kantar et al. 2019; Liu et al. 2021). Lü et al. (2019) found that pyrite activated the ZVI surface through suppressing pH increase in the reactor and replacing the Fe-oxide layer with FeS on the ZVI surface. Similarly, Linting et al. (2021) informed that pyrite, not only, stimulated ZVI corrosion, but also, increased iron redox cycling to produce more Fe(II) sites on the ZVI surface. The authors also documented the activation of ZVI by pyrite, resulting in the formation of iron sulfide (FeS) on the ZVI surface. This process expedites the passage of electrons from ZVI to the surface-bound species, such as contaminants and Fe^3+^, due to the superior electron conducting capability of FeS compared to surface oxidation species. Min et al. (2021) stated that the particles prepared through the ball milling of ZVI and pyrite exhibited a significant chromium reductive ability through surface sulfidation of ZVI by pyrite. Using bentonite-supported ZVI as the catalyst, Diao and Chu (2021) found that the presence of pyrite enhanced the Fenton oxidation of atrazine through the enhanced corrosion of ZVI particles and solution pH suppressing.

Although pyrite has been shown to enhance the adsorptive and reductive ability of ZVI for the enhanced removal of contaminants such as As, Cr(VI), and nitrobenzene (e.g., Du et al. 2019; Linting et al. 2021), the data regarding the effect of pyrite on CP removal with the Fenton process employing micron-sized ZVI as the catalyst is scarce in the literature. Few studies have, primarily, focused on CP removal using either pyrite or ZVI as the sole catalyst in the heterogeneous Fenton process (e.g., Ezzatahmadi et al. 2019; Xu et al. 2019; Kantar et al. 2019; Lv et al. 2024). In batch and column studies performed with a mixture of ZVI and pyrite, Oral et al. (2024a, b) revealed that the removal of organic contaminants such as diclofenac and methylene blue was a complex phenomenon, involving sequentially and/or concurrently occurring several different processes such as adsorption, chemical precipitation, and degradation. However, here we show that the CP removal by the ZVI/pyrite system in the presence of H_2_O_2_ was primarily dominated by oxidative degradation of CPs with some strong radicals (e.g., •OH) in the solution and on the catalyst surface. Hence, the aims of this current study were to (1) further elucidate the role of pyrite on the removal of chlorophenols (CP) by the ZVI/H_2_O_2_ system under varying chemical conditions, such as different doses of pyrite and ZVI, (2) assess the reaction mechanisms involved in the oxidative degradation of CPs by the ZVI/pyrite/H_2_O_2_ system, and (3) evaluate the biodegradability of CP intermediates following Fenton degradation. All experiments used 4-chlorophenol (4-CP) and 2,4-dichlorophenol (2,4-DCP) as representative chlorophenolic compounds in the study since these two CPs are commonly observed in industrial wastewaters at ppm levels (Eslami et al. 2018; Kantar et al. 2019).

## Materials and methods

### Materials and chemicals

All chemicals employed in the study had reagent grade or higher quality. The solutions for 4-CP and furfuryl alcohol (FA) were supplied by Sigma-Aldrich. All other chemicals, including 2,4-DCP, H_2_O_2_, methanol, tert-butanol (TBA), p-benzoquinone (BQ), n-hexane, iron(II) sulfate heptahydrate, acetonitrile, and 1,1-phenanthroline monohydrate were obtained from Merck. Pyrite was supplied by a flotation plant as pyrite concentrate in Turkey. The pyrite concentrate contained > 95% pyrite and some silicate minerals. The X-ray diffraction (XRD) spectra of pyrite concentrate are given in Fig. S1. The typical peaks of pyrite were observed at 2*θ* = 28.74°, 33.26°, 37.29°, 40.96°, 47.64°, 56.48°, 59.18°, 61.8°, and 64.44°. As shown in the scanning electron microscopy (SEM) image (Fig. S2a), the pyrite concentrate contained particles (40 µm in size) with rough surfaces containing some smaller particles attached. The pyrite concentrate was directly used in the experiments without any further treatment. The electron-dispersive spectroscopy (EDS) spectra show peaks related to elements such as Fe, S, Si, and O, confirming the presence of oxidized species of Fe and silicates on the pyrite surface (Fig. S2b). Zero-valent iron (ZVI) was purchased from Merck, and activated with 0.1 M hydrochloric acid prior to the experiments. The XRD peaks observed at 2*θ* = 44.84° and 65.17° are the typical peaks of ZVI particles (Fig. S3).

### Experimental procedure

Batch degradation experiments were implemented in 250 mL glass reactors at room temperature. The working solution had a volume of 200 mL, and contained a desired concentration of H_2_O_2_ (0–0.02 M). The optimum H_2_O_2_ concentration (0.005 M) was determined by performing degradation experiments under variable H_2_O_2_ concentrations as given in Fig. S4. In all experiments, the initial 4-CP or 2,4-DCP concentration was adjusted to 100 mg L^−1^, which was close to the concentration of CPs detected in actual pharmaceutical wastewaters (Eslami et al. 2018). The initial solution pH was 4.8 and 5.2 for 4-CP and 2,4-DCP, respectively, and no pH adjustment was performed during the experiments to assess the pH regulating ability of pyrite for optimum Fenton operation in batch reactors. All batch experiments were carried out at variable pyrite/ZVI concentrations ranging from 0 to 1 g L^−1^. The reaction was initiated when the solids were mixed with the liquid in the reactor. The reactor was shaken on a shaker at 250 rpm, and samples taken at desired sampling times were immediately spiked with 100 µL 0.1 M TBA, and filtered through a 0.45-µm syringe filter.

To determine reaction mechanisms and the types of radicals involved in the oxidative treatment of 4-CP and 2,4-DCP, the reactors were spiked with 0.01 M TBA as hydroxyl radical (•OH) scavenger, 1 mM BQ as superoxide radical ($\bullet {\text{O}}_{2}^{-}$) scavenger, and 0.01 M FA as singlet oxygen (^1^O_2_) scavenger (Liu et al. 2023). The reproducibility of data was ensured by performing duplicate experiments. The data from the batch experiments were used to derive first-order reaction kinetic parameters using the following equation (Kantar et al. 2019):

1 $$\text{ln}\left[\text{C}\right]=\text{ln}\left[{\text{C}}_{\text{o}}\right]+\text{kt}$$

where *k* represents the reaction rate constant (min^−1^), and *C*_o_ describes the initial 4-CP or 2,4-DCP concentration.

### Surface characterization

The elemental and mineral composition, functional groups, surface morphology, and surface oxidation products of solid samples obtained from batch reactors at desired times were investigated by Fourier transform infrared (FTIR) spectroscopy (Thermo Nicolet-iS10), XRD (PANalytical Empyrean), SEM–EDS (JEOL SEM-7100-EDX), and X-ray photoelectron spectroscopy (XPS) (PHI 5000 Versa Probe). The samples were taken from the batch reactors once all CPs were completely depleted in the reactor. The samples were then centrifuged at 5600 rpm for 10 min, and dried in an oven at 60 °C for 1 h prior to the measurements. Salt titration experiments were carried out to investigate the surface charge variation on suspension samples withdrawn from the reactor at desired times (Oral et al. 2024a). The surface areas (*S*_A_) of solid materials obtained from the batch reactors before and after Fenton oxidation were determined by BET method (Oral et al. 2024a). A detailed explanation for these measurements can be found in Supporting Information (Page S6).

### Analytical methods

The concentrations of organic acids, benzoquinone, hydroquinone, chlorohydroquinone, 4-CP, and 2,4-DCP were determined with high-pressure liquid chromatography (HPLC). The working conditions for HPLC analyses are given in Table S1. The evolution of intermediate reaction by-products was monitored by GC–MS measurements (Kantar et al. 2019). The samples from batch degradation experiments were analyzed for their ferrous iron and total iron contents using spectrophotometric 1,10 phenanthroline method and atomic absorption spectrophotometer, respectively. The total organic carbon (TOC) concentrations were evaluated with a total organic carbon analyzer. Chemical oxygen demand (COD) and biological oxygen demand (BOD) measurements were performed according to experimental methods given by Kayan et al. (2021). The aqueous chloride contents were determined spectrophotometrically with mercuric thiocyanate method (Kantar et al. 2019). Detailed information on analytical procedures is supplied in Supporting Information (Page S7-9).

## Results and discussion

### Role of pyrite/ZVI concentration on CP removal

**Fig. 1:**
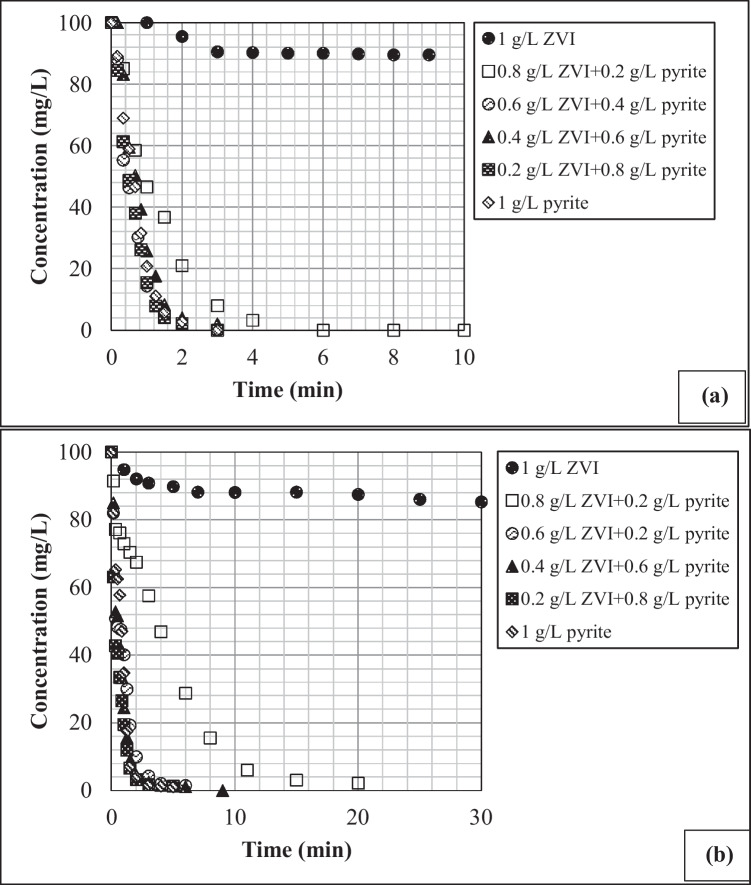
The role of variable pyrite/ZVI doses on chlorophenol degradation by ZVI-Fenton process in the presence of** a** 4-CP (initial pH = 4.8, H_2_O_2_ = 0.005 M; 4-CP = 100 mg L^−1^) and **b** 2,4-DCP (initial pH = 5.2; H_2_O_2_ = 0.005 M; 2,4-DCP = 100 mg L^−1^)

The effects of varying pyrite/ZVI concentration on the removal of 4-CP and 2,4-DCP by Fenton oxidation are presented in Fig. 1a and b, respectively. The data given in Fig. 1 were modeled with a first-order rate equation (Eq. 1) and the model parameters are given in Tables S2 and S3 for 4-CP and 2,4-DCP, respectively. As shown in Fig. 1, the reactor with 1 g L^−1^ ZVI as the sole catalyst in the Fenton process had the lowest removal efficiency for both 4-CP and 2,4-DCP. However, the catalytic activity of ZVI increased significantly by adding pyrite to the reactor. The highest 4-CP and 2,4-DCP removal was achieved at a mixture of 0.8 g L^−1^ pyrite and 0.2 g L^−1^ ZVI. Hereafter, this pyrite and ZVI dose will be mentioned as “optimum pyrite/ZVI concentration” in the text. However, the CP removal efficiency for both 4-CP and 2,4-DCP decreased slightly at pyrite concentration greater than 0.8 g L^−1^. A number of studies performed, primarily with metal ions such as chromium, demonstrated that if mixed with ZVI at an optimum dose, pyrite could increase metal ion removal by ZVI through enhanced surface corrosion of ZVI (Du et al. 2019; Oral et al. 2024a, b).

Figure S5 shows that the CP degradation was closely related to Cl^−^ discharge from the aromatic structure at low reaction times for both 4-CP and 2,4-DCP. This result implies that the CP removal was initially driven by the hydroxyl radical attack on aromatic structure, breaking the C–Cl bond, and thus discharging Cl^−^ ion into the solution for both 4-CP and 2,4-DCP. A study performed by Kantar et al. (2019) reported a similar finding for the Fenton degradation of CPs using pyrite as the sole catalyst. However, at higher reaction times, surprisingly, in system with 4-CP (Fig. S5a), the Cl^−^ concentration was observed to decrease at time > 50 s, suggesting that Cl^−^ may adsorb onto the solid or may be complexed with Fenton oxidation intermediate species. Considering the negative surface charge associated with pyrite under acidic conditions (pH > 2), it is more likely that the Cl atoms released from the aromatic structures of CPs became associated with other chlorinated Fenton oxidation species since the data from literature show that Fenton oxidation usually transforms CPs into some intermediate products, including chlorinated organic compounds (Kantar et al. 2019). Similarly, in system with 2,4-DCP (Fig. S5b), the 2,4-DCP degradation increased in parallel with Cl^−^ release at *t* < 1 min, and then reached 100%. However, at higher reaction times, the Cl^−^ release remained only at 92%, suggesting that the 2,4-DCP removal at higher reaction times was highly influenced by some additional processes, including the adsorption of 2,4-DCP onto solid surfaces (Fig. S5b). As shown in Fig. S6, while the CP removal for both 4-CP and 2,4-DCP reached 100% within a couple of minutes in the presence of H_2_O_2_, it only remained at 10 to 25% in the absence of H_2_O_2_, depending on the type of CP studied. This confirms that the CP removal was initially controlled by oxidative degradation by the pyrite/ZVI/H_2_O_2_ system, and the adsorption of CPs onto pyrite/ZVI particles had negligible effect on CP removal (i.e., 10–25%) at reaction times less than 5 min (Fig. S6a and b) since the experiments involved micron-sized pyrite and ZVI particles with very low surface areas (Oral et al. 2024a). In a study conducted with different chlorophenols, it was revealed that the adsorption of CPs onto pyrite occurred through hydrophobic bonding, and increased with increasing the number of Cl atoms in the aromatic structure due to enhanced hydrophobic characteristics (Kantar et al. 2019).

Figure S7 shows the role of radical scavengers on 4-CP and 2,4-DCP removal. While BQ had a negligible impact on 4-CP and 2,4-DCP degradation, both FA and TBA drastically lowered CP degradation compared to non-scavenger-containing control experiments (Fig. S7a and b). This suggests that the oxidative degradation of both 4-CP and 2,4-DCP was mainly governed by •OH and ^1^O_2_ radicals. This result agrees well with the findings of Bao et al. (2020) and Liu et al. (2023) who stated that the superoxide radical (•${\text{O}}_{2}^{-})$ was very unstable in the process, and was rapidly transformed into ^1^O_2_ during Fenton degradation of organic contaminants such as 4-CP and reactive dyes. However, some additional spectroscopic measurements are needed to better clarify the influence of these radicals on CP removal by the pyrite/ZVI/H_2_O_2_ system.

### Role of pyrite on pH control and Fe dissolution

**Fig. 2:**
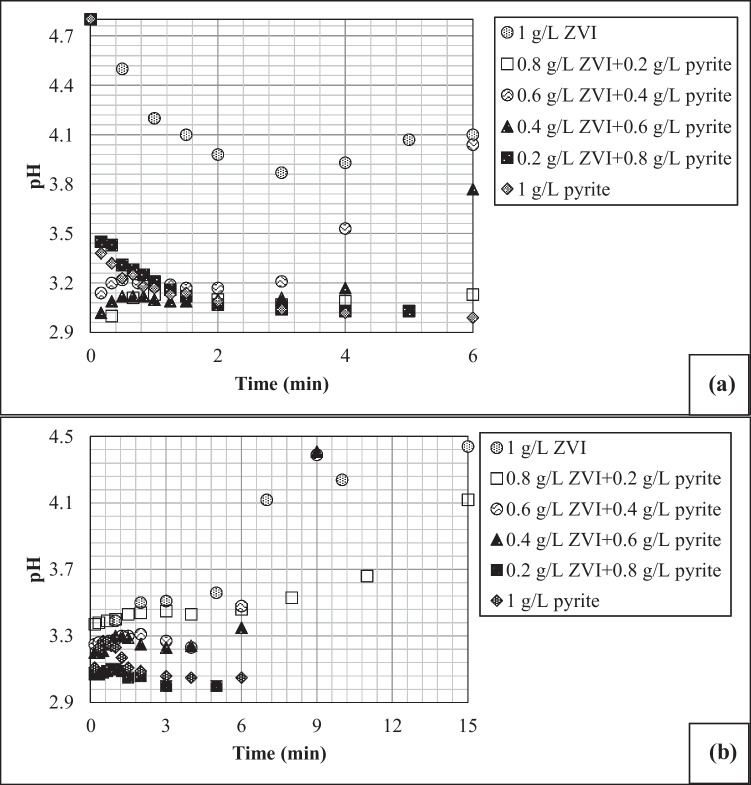
The role of variable pyrite/ZVI doses on pH variation in batch reactors containing **a** 4-CP (H_2_O_2_ = 0.005 M; 4-CP = 100 mg L^−1^) and **b** 2,4-DCP (H_2_O_2_ = 0.005 M; 2,4-DCP = 100 mg L^−1^)

It has been shown that Fenton reactions require acidic conditions (pH 2–4) and enhanced Fe redox cycling for a sustainable Fenton operation (Kantar et al. 2019; Liu et al. 2021; Chen et al. 2023; Zhang et al. 2023; Zhou et al. 2023; Oral et al. 2024a). According to data from a number of studies, the ZVI surface becomes passivated with increasing solution pH due to the low solubility and the development of Fe-oxide layer on the ZVI surface, especially at pH > 4 (Bao et al. 2020; Kong et al. 2021; Hu et al. 2021). As stated above, to understand the role of pyrite on pH control, the batch experiments were conducted at the initial pH values of CP solutions (pH 4.8 for 4-CP and pH 5.2 for 2,4-DCP) with no further pH arrangements until the end of the experiments. Figure 2 shows the extent of pH variation in reactors operated at different pyrite and ZVI doses with 100 mg L^−1^ 4-CP (Fig. 2a) and 100 mg L^−1^ 2,4-DCP (Fig. 2b). It is clear that while in the system with no pyrite (i.e., 1 g L^−1^ ZVI), the suspension pH remained above pH 3 throughout the experiments for both 4-CP and 2,4-DCP. This explains the lowest 4-CP and 2,4-DCP removals in systems with 1 g L^−1^ ZVI as the sole catalyst. However, as presented in Fig. 2, increasing the pyrite dose in the reactor lowered the suspension pH below 3, which, in turn, led to an increase in CP removal efficiency for both 4-CP and 2,4-DCP, as depicted in Fig. 1. The decline observed in pH with increasing pyrite concentration is not surprising since pyrite oxidation with O_2_ and H_2_O_2_ generates protons as follows (Liu et al. 2023):

2 $$2{\text{FeS}}_{2}+7{\text{O}}_{2}+{2\text{H}}_{2}\text{O}\to 2{\text{Fe}}^{2+}+4{\text{H}}^{+}+4{\text{SO}}_{4}^{2-}$$

3 $$2{\text{FeS}}_{2}+15{{\text{H}}_{2}\text{O}}_{2}\to 2{\text{Fe}}^{3+}+14{\text{H}}_{2}\text{O}+2{\text{H}}^{+}+4{\text{SO}}_{4}^{2-}$$

The acidic conditions accelerate the dissolution of Fe-oxide surface species from ZVI and pyrite, thus eliminating or minimizing the formation of a passivating layer on the catalyst surface (Lü et al. 2019; Linting et al. 2021). For example, it has been reported in the literature that increasing pyrite dose drastically stimulated atrazine degradation by bentonite-supported ZVI because of the release of sulfuric acid into solution (Diao and Chu 2021). Similarly, Hu et al. (2021) stated that the presence of pyrite significantly increased Fe solubility from ZVI by decreasing solution pH to more acidic values. These authors also report that lowering solution pH to more acidic values by adding pyrite to the system led to a decrease in co-precipitation and adsorption of dyes relative to single ZVI system. The Fe^2+^ species dissolved from pyrite/ZVI particles as a result of Reactions (2, 4, and 5) could, in turn, react with H_2_O_2_ to generate •OH radicals in the aqueous phase and/or on the catalyst surface (Reaction 6):

4 $${\text{Fe}}^{0}+{2\text{H}}_{2}\text{O}\to {\text{Fe}}^{2+}+{\text{H}}_{2}+2{\text{OH}}^{-}$$

5 $${\text{Fe}}^{0}+{\text{H}}_{2}{\text{O}}_{2}+2{\text{H}}^{+}\to {\text{Fe}}^{2+}+2{\text{H}}_{2}\text{O}$$

6 $${\text{Fe}}^{2+}+{\text{H}}_{2}{\text{O}}_{2}\to {\text{Fe}}^{3+}+ \bullet \text{OH}+{\text{OH}}^{-}$$

**Fig. 3:**
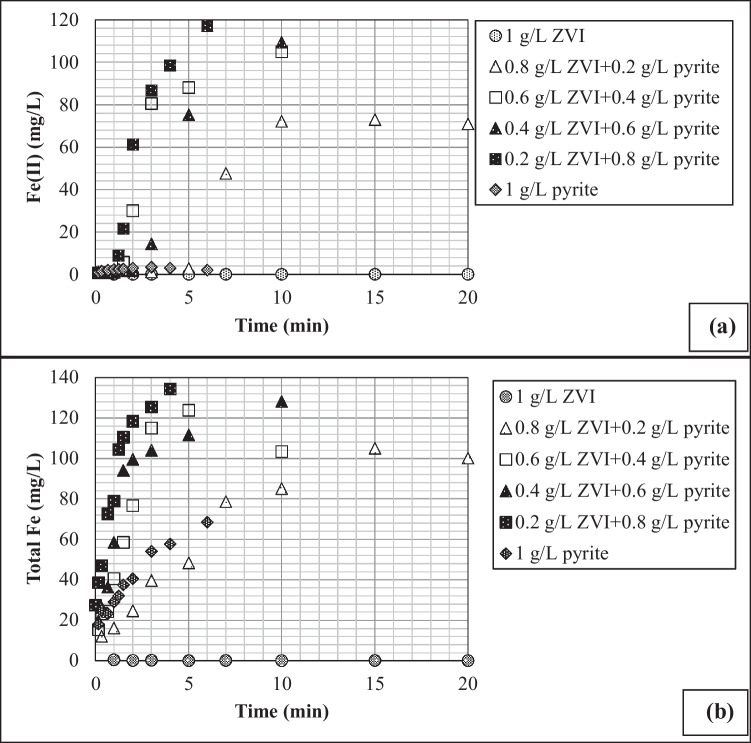
The role of variable pyrite/ZVI dose on Fe leaching in batch reactors with 100 mg L^−1^ 2,4-DCP, 0.005 M H_2_O_2_, and an initial pH of 5.2 for **a** Fe(II) and **b** total Fe

Figure 3 shows that while, in the system with ZVI as the sole catalyst (i.e., 1 g L^−1^ ZVI), the Fe(II)/total iron leaching was very limited, the addition of pyrite significantly increased solution phase Fe(II) (Fig. 3a) and total iron (Fig. 3b) concentrations in the system containing 2,4-DCP. As depicted in Fig. 2b, the suspension pH remained at the highest value in the absence of pyrite, and thus the Fe species accumulated on the ZVI surface due to their low solubility under a slightly acidic to neutral pH environment, thereby, resulting in the loss of reactivity of ZVI due to surface passivation (Li et al. 2015; Ezzatahmadi et al. 2019; Liu et al. 2021; Guan et al. 2022). Li et al. (2015) stated that the accumulation of Fe-oxides on zero-valent iron surface hindered Fe leaching from the ZVI surface. Of all the pyrite and ZVI concentrations tested, the highest Fe(II) and total iron concentrations were measured in systems with an optimum pyrite/ZVI concentration (Fig. 3a, b). This overlaps well with the highest CP degradation rates as given in Fig. 1. Figure 3a shows that the Fe^2+^ concentration was very high in batch reactor with 2,4-DCP, implying that homogenous Fenton reaction also played a major role on 2,4-DCP degradation (Reaction 6). High Fe dissolution in systems with increasing pyrite dose indicates that the acidic conditions created by pyrite led to the production of new active sites on ZVI and pyrite surfaces through enhanced Fe redox cycling (Zhang et al. 2023; Chen et al. 2023; Zhou et al. 2023; Oral et al. 2024a, b):

7 $${\text{FeS}}_{2}+14{\text{Fe}}^{3+}+8{\text{H}}_{2}\text{O}\to 15{\text{Fe}}^{2+}+16{\text{H}}^{+}+2{\text{SO}}_{4}^{2-}$$

8 $$2{\text{Fe}}^{3+}+{\text{Fe}}^{0}\to 3{\text{Fe}}^{2+}$$

The enhanced Fe dissolution with pyrite addition is in good agreement with the findings of Du et al. (2020), who reported that pyrite sustained the reactivity of ZVI by achieving more acidic conditions and accelerating ZVI corrosion relative to single ZVI system. Similarly, Du et al. (2019) demonstrated that, because of its relatively high positive standard potential, pyrite could function as a cathode, and mediate electron transfer from ZVI (Fe^0^) core to O_2_ and/or Fe^3+^, thus inhibiting or delaying the deposition of Fe-oxide film and subsequently regenerating new Fe(II) sites on the ZVI surface. According to several authors, on the other hand, the positive impact of pyrite on enhancing ZVI catalytic activity in the Fenton reaction was also attributed to the replacement of Fe-oxide film on the ZVI surface by FeS_x_, which exhibits much better electron conducting ability to speed up the electron passage from ZVI to the sorbed surface compounds, including Fe^3+^ (Lü et al. 2019). Using some spectroscopic data, Lü et al. (2019), for instance, showed that the sulfate ions released from pyrite (Reactions 2, 5, and 7) could oxidize ZVI to form Fe^2+^ and HS^−^, followed by the formation of FeS on ZVI surface via the interaction of Fe^2+^ with HS^−^.

### Surface characterization

**Fig. 4:**
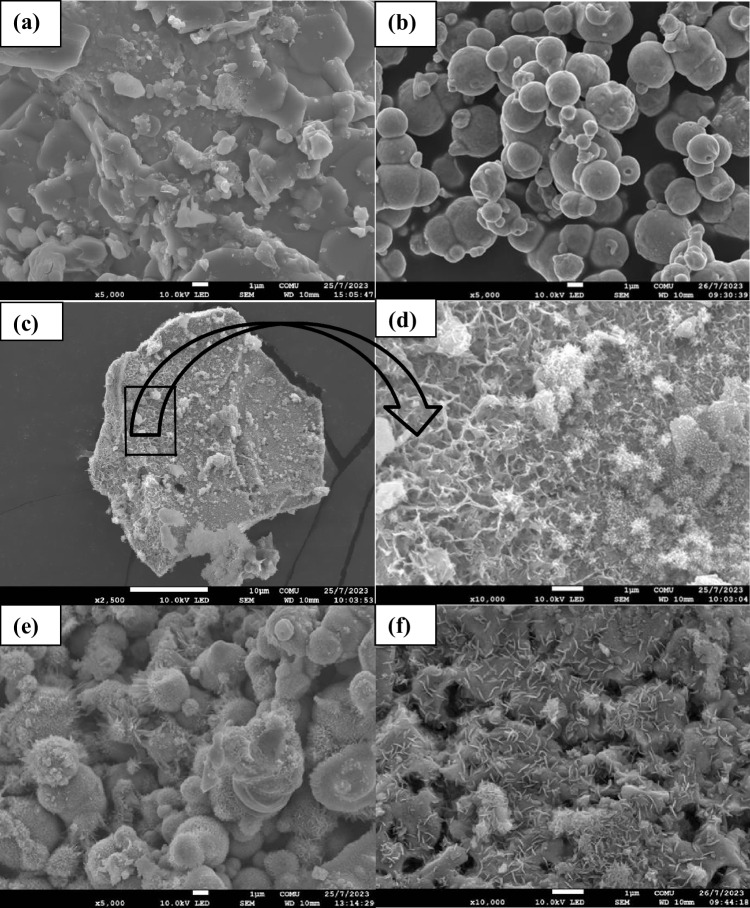
SEM photographs of solid samples taken from batch reactors containing **a** 1 g L^−1^ pyrite and 0.005 M H_2_O_2_, **b** 1 g L^−1^ ZVI and 0.005 M H_2_O_2_, **c**, **d** 0.2 g L^−1^ ZVI + 0.8 g L^−1^ pyrite and 0.005 M H_2_O_2_, and **e**, **f** 0.2 g L^−1^ ZVI + 0.8 g L^−1^ pyrite, 100 mg L^−1^ 2,4-DCP, and 0.005 M H_2_O_2_

Figure 4 shows that the particles withdrawn from the reactors containing 1 g L^−1^ pyrite and 0.005 M H_2_O_2_ exhibited rough surfaces with no significant accumulation of surface oxidation products (Fig. 4a). As seen in Fig. 4b, the SEM photographs of the solid materials withdrawn from the reactor with 1 g L^−1^ ZVI and 0.005 M H_2_O_2_ provided evidence for the agglomeration of spherical ZVI particles in the reactor. In a system with 0.005 M H_2_O_2_ and an optimum pyrite/ZVI dose (Fig. 4c and e), however, the pyrite particles were observed to be covered by some surface oxidation products (i.e., Fe-oxides/sulfides) and ZVI particles in the absence or presence of 2,4-DCP. The surface precipitates, deposited on pyrite and ZVI surface, were composed of needle-shaped crystals (Fig. 4d and f), confirming a major surface corrosion of ZVI particles during the Fenton reaction (Oral et al. 2024a). This type of surface precipitate was attributed to the generation of green rust sulfate in the literature (Lü et al. 2019). The EDS spectra show that, compared to a single pyrite system (Fig. S8a), while the Fe content of composite solid samples with an optimum pyrite/ZVI concentration was observed to increase, the S content decreased significantly (Fig. S8b). The EDS spectra of the composite sample taken in the reactor with an optimum pyrite/ZVI concentration, 0.005 M H_2_O_2_, and 100 mg L^−1^ 2,4-DCP showed a small peak related to Cl, implying the presence of 2,4-DCP and/or other chlorinated reaction by-products on the solid surface (Fig. S8c).

The XRD spectra of the composite material withdrawn from the system with an optimum pyrite/ZVI dose and 0.005 M H_2_O_2_ displayed XRD peaks that overlapped with the peaks of raw pyrite and ZVI, thus confirming the presence of ZVI and pyrite particles in the composite sample (Fig. S9). However, the peak belonging to ZVI at 2*θ* = 65.17° disappeared from the spectra (Fig. S9), providing further evidence for ZVI surface corrosion and/or deposition of amorphous surface species containing FeS (Min et al. 2021).

**Fig. 5:**
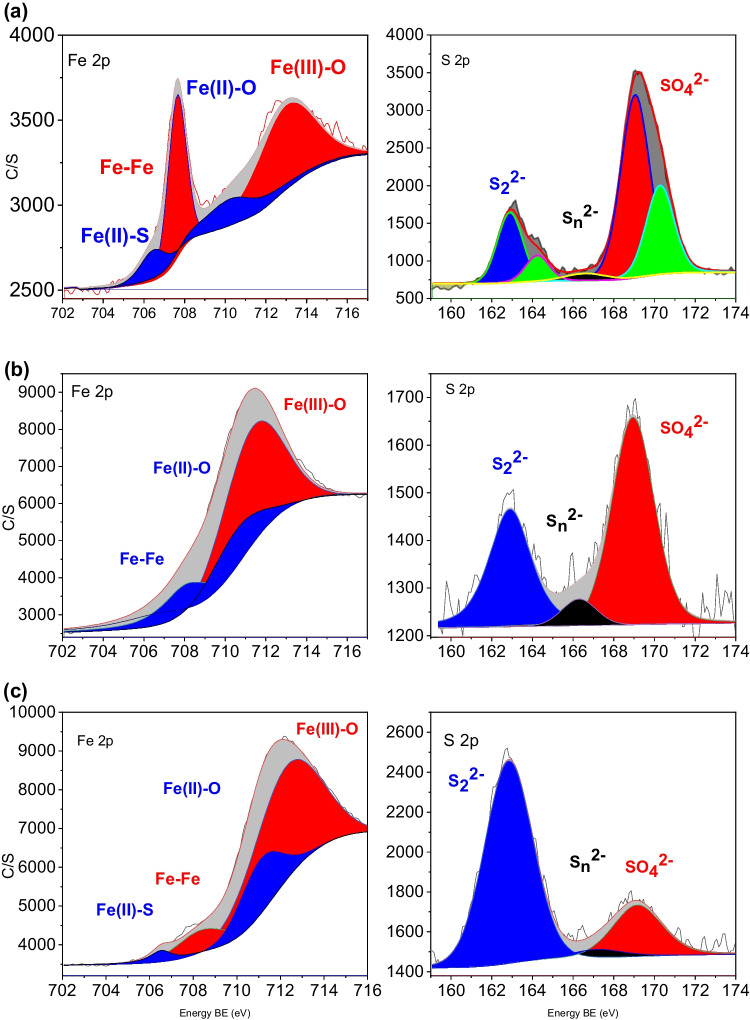
Fitted Fe 2p and S 2p X-ray photoelectron spectra (XPS) of **a** raw pyrite, **b** 0.2 g L^−1^ ZVI + 0.8 g L^−1^ pyrite + 0.005 M H_2_O_2_, and **c** 0.2 g L^−1^ ZVI + 0.8 g L^−1^ pyrite + 0.005 M H_2_O_2_ + 100 mg L^−1^ 2,4-DCP

Figure 5 shows the Fe 2p and S 2p XPS spectra for samples taken from the batch reactors. The Fe 2p spectra for the solid particles taken from the system with 1 g L^−1^ pyrite showed peaks at 706.48 eV for Fe(II)-S, 710.23 eV for Fe(II)-O, and 713.01 eV for Fe(III)-O species, respectively (Fig. 5a). Similarly, the S 2p spectra for the same sample included peaks at 162.89 eV for disulfide (S_2_^2−^), 164.26 eV for polysulfides, and 169.05 to 170.28 eV for sulfate (Fig. 5a). The presence of Fe(III)-O and sulfate species revealed that the raw pyrite surface was partially oxidized. As shown in Fig. 5b, the XPS Fe 2p spectra for samples with an optimum pyrite/ZVI dose and 0.005 M H_2_O_2_ in the absence of 2,4-DCP displayed Fe(II)-S peak at 706.68 eV, Fe peak at 708.10 eV, Fe(II)-O peak at 710.45 eV, and Fe(III)-O peak at 711.32 eV, implying the surface oxidation of ZVI by H_2_O_2_ (Reaction 5). The S 2p spectra of the same sample contained peaks at 162.9 eV and 168.94 eV, corresponding to disulfide and sulfate, respectively (Fig. 5b). The comparison of areas under the XPS peaks indicated that, compared to raw pyrite samples (Fig. 5a), the solid particles withdrawn from the system with an optimum pyrite/ZVI dose and 0.005 M H_2_O_2_ was richer in Fe, but lower in S contents (Fig. 5b). This correlates well with the results of SEM–EDS measurements which provide evidence for the masking of the pyrite surface by surface Fe precipitates and ZVI particles (Fig. 4c and e). The presence of Fe(III)-O peak at 712.25 eV and sulfate peak at 169.15 eV for the composite pyrite/ZVI sample presented evidence that the surface oxidation products still existed on the solid samples in the presence of 100 mg L^−1^ 2,4-DCP (Fig. 5c). However, the composite sample obtained from the batch system with an optimum pyrite/ZVI concentration, 100 mg L^−1^ 2,4-DCP, and 0.005 M H_2_O_2_ had much less O, but much higher disulfide contents compared to the composite sample with no 2,4-DCP (Fig. 5b and c), implying the interaction of 2,4-DCP and/or its degradation by-products with ZVI and surface precipitates.

**Fig. 6:**
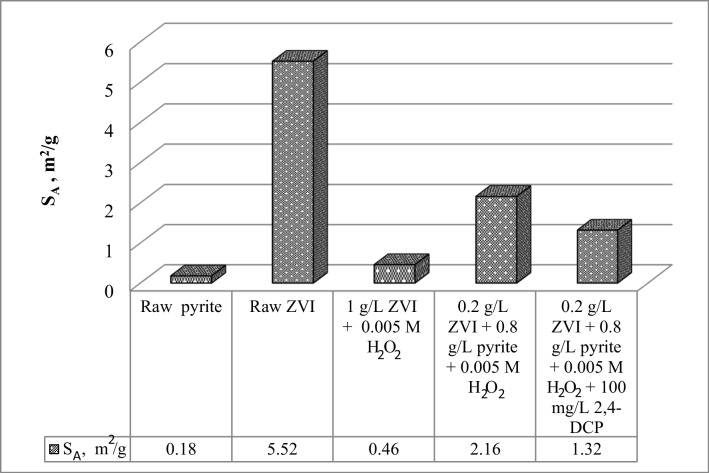
Comparison of BET surface areas (*S*_A_) of solid particles obtained under different experimental conditions

**Fig. 7:**
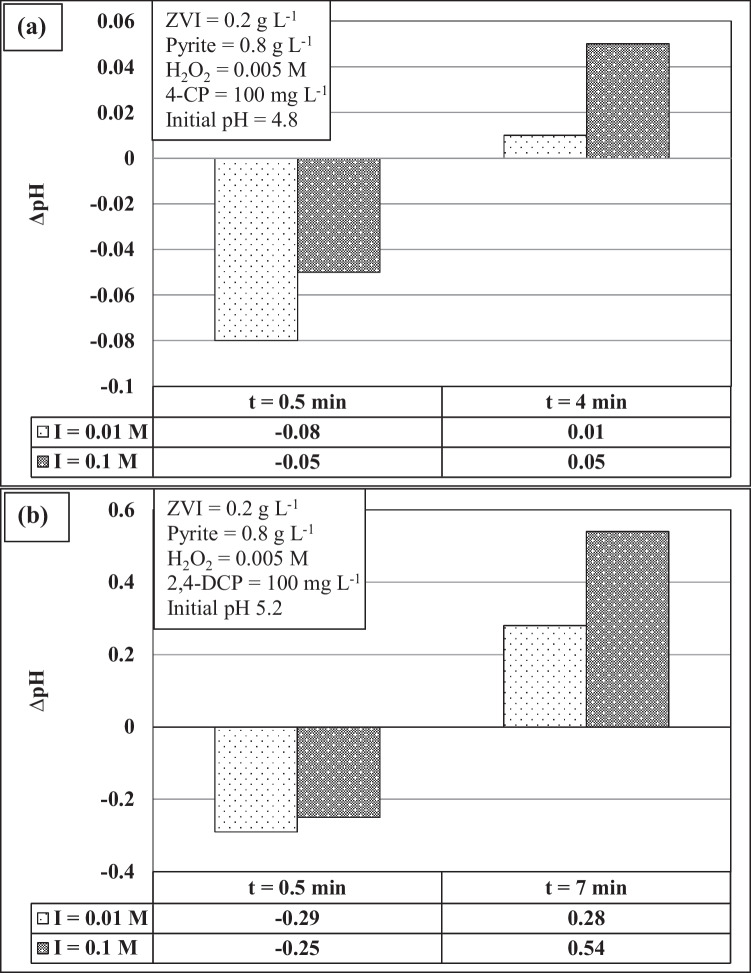
Surface charge variation of solid particles withdrawn from the batch reactors at different reaction times for **a** 4-CP and **b** 2,4-DCP

The BET surface area (*S*_A_) measurements show that the *S*_A_ of the ZVI sample withdrawn from the system with 1 g L^−1^ ZVI and 0.005 M H_2_O_2_ was measured to be much lower relative to raw zero-valent iron particles (Fig. 6). This is indicative of ZVI particle agglomeration in the batch reactor due to dipole–dipole interaction (Bao et al. 2020), as also confirmed by SEM photographs shown in Fig. 4b. However, the composite sample obtained from the batch system containing a mixture of pyrite/ZVI particles at optimum concentration exhibited much higher *S*_A_ (2.16 m^2^ g^−1^) compared to the reactor with 1 g L^−1^ ZVI. This implies that the particle aggregation was significantly reduced as a result of ZVI particle deposition onto the pyrite surface. The salt titration experiments conducted with the samples withdrawn from the systems with an optimum pyrite/ZVI dose and 100 mg L^−1^ 4-CP or 2,4-DCP show that while the suspension particles were initially negatively charged at time, *t* = 30 s, the surface charge of the particles changed from negative to positive values at higher reaction times (Fig. 7a, b). In good agreement with the SEM photographs and XPS results (Fig. 4 and Fig. 5), the change in surface charge may be explained through the masking of the negatively charged pyrite surface with positively charged ZVI and/or surface Fe-oxides/sulfides. As shown in Fig. 6, the solid materials withdrawn from the system with an optimum pyrite/ZVI dose and 100 mg L^−1^ 2,4-DCP had a particle *S*_A_ (1.32 m^2^ g^−1^) lower than that measured for the composite particle in the absence of 2,4-DCP (2.16 m^2^ g^−1^), implying that a portion of surface Fe-oxide/sulfide precipitates was washed away by the action of 2,4-DCP degradation intermediate species, including low molecular weight organic acids.

**Fig. 8:**
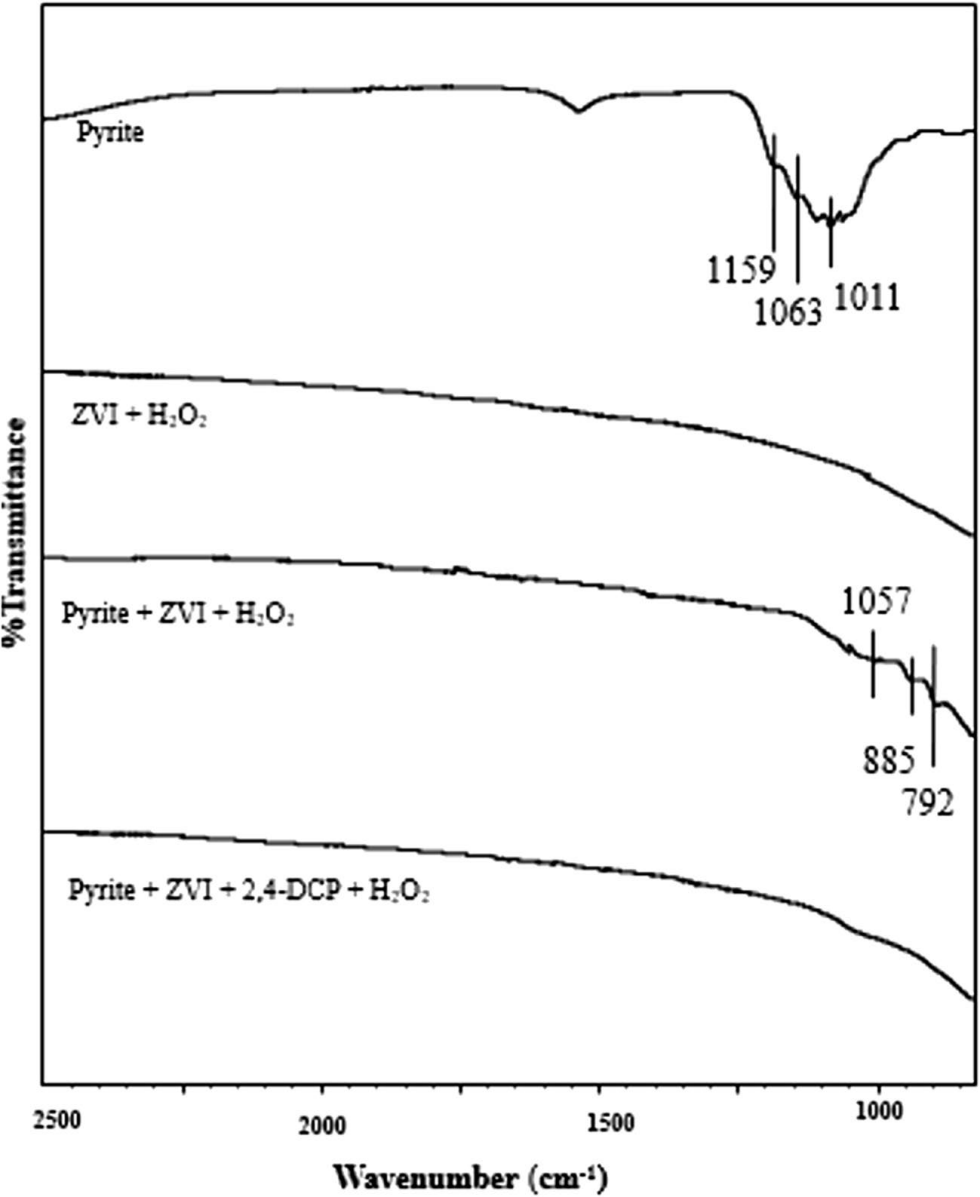
FTIR spectra of particles withdrawn from the batch experiments  performed at  different system conditions

Figure 8 presents the FTIR spectra of different solid samples taken from batch reactors. The solid samples obtained from the reactor with 1 g L^−1^ pyrite exhibited peaks at 1011 and 1159 cm^−1^, corresponding to ferric/ferrous sulfate species (Reyes-Bozo et al. 2015; Oral et al. 2024a). The peak at 1038 cm^−1^ was attributed to the presence of a thin layer of ϒ-FeOOH surface oxidation product (Reyes-Bozo et al. 2015; Sulaiman and Al-Jabari 2021; Tarekegn et al. 2021). The peak at 1063 cm^−1^ was correlated to the double bond S = S stretching vibration from FeS_2_ (Sun et al. 2017; Oral et al. 2024a). The peak at 1647 cm^−1^ was related to the adsorbed water molecule (Oral et al. 2024a). On the other hand, consistent with the study of Su et al. (2018), the ZVI samples withdrawn from the batch system containing 1 g L^−1^ ZVI and 0.005 M H_2_O_2_ did not exhibit any specific peak (Oral et al. 2024a). Similarly, the FTIR spectra of the solid samples withdrawn from the reactor with an optimum pyrite/ZVI concentration and 0.005 M H_2_O_2_ showed that most of the peaks observed in the pyrite sample disappeared or shifted positions, implying that the pyrite surface behaved more like a ZVI surface (Oral et al. 2024a). Sun et al. (2017) reported that the peaks detected at around 885 cm^−1^ and 792 cm^−1^ were linked to the formation of iron sulfide species. On the other hand, the FTIR spectra showed a peak shift from 1063 to 1057 cm^−1^ in the composite pyrite/ZVI sample, implying the interaction of zero-valent iron particles and/or surface oxidation species with the disulfide group of pyrite (Oral et al. 2024a). Surprisingly, the sample withdrawn from the reactor with an optimum pyrite/ZVI concentration, 0.005 M H_2_O_2_, and 100 mg L^−1^ 2,4-DCP did not exhibit any major peak. This indicates that the CP molecules adsorbed onto the catalyst surface were rapidly oxidized by surface •OH radicals and then rapidly released back into the solution.

### Degradation intermediates and their biodegradability

Figure S10 shows that while the 4-CP and 2,4-DCP degradation was completed within a couple of minutes, the TOC removal was very slow, and ranged only from 20 to 40%, depending on the type of CP studied. This indicates that Fenton oxidation led to the conversion of both 4-CP and 2,4-DCP into some new organic species. As shown in Fig. S5, the release of Cl^−^ ion was paralleled with CP degradation, providing further evidence for the breakdown of the C–Cl bond on the aromatic benzene structure by •OH radical attack. According to Du et al. (2007) and Liu et al. (2015), the •OH radical could attack C-H and C–Cl bonds in the aromatic 4-CP structure, thus releasing hydroquinone (HQ), benzoquinone (BQ), 4-chlorocatechol, and some aliphatic organic acids. Similarly, according to the findings of Wang et al. (2017) and Kantar et al. (2019), the primary by-products resulting from the breakdown of 2,4-DCP through the Fenton process consisted of phenol, catechol, hydroquinone (HQ), benzoquinone (BQ), and chlorohydroquinone (CHQ) as well as acetic and formic acids. Figures S11 and S12 show the generation of major aromatic compounds and organic acids during Fenton degradation of 4-CP and 2,4-DCP in reactors with an optimum pyrite/ZVI dose, and 0.005 M H_2_O_2_. Of all these aromatic organic molecules (e.g., BQ, CHQ, HQ) and organic acids (e.g., acetic acid, formic acid) analyzed, BQ, acetic acid, and formic acid were observed to be the most dominant species during Fenton treatment of 4-CP and 2,4-DCP. In addition, other aromatic compounds such as phenol, 1,3,5-trimethylbenzene and 3,4-dichlorocatechol were also detected in samples with 2,4-DCP (data not shown).

**Fig. 9:**
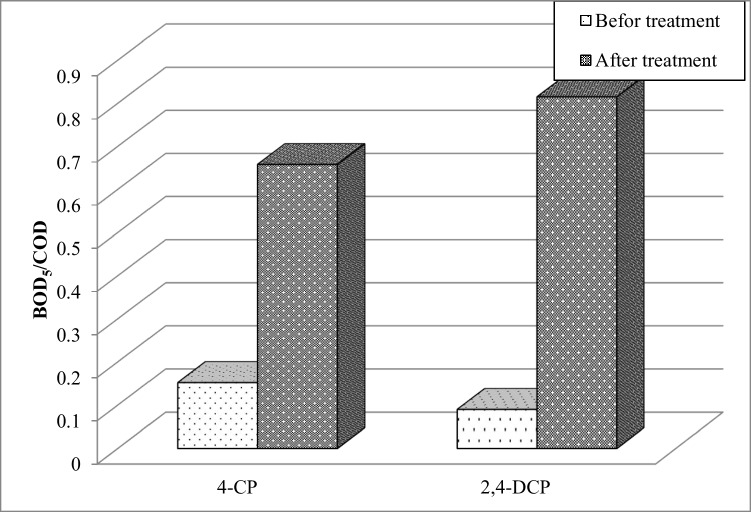
Comparison of biodegradability of untreated chlorophenols and their Fenton degradation intermediate products. The Fenton degradation was achieved using 0.2 g L^−1^ ZVI and 0.8 g L^−1^ pyrite. The H_2_O_2_ concentration was set to a value of 0.005 M for all degradation experiments

Figure 9 shows the degree of biodegradability of 4-CP and 2,4-DCP degradation intermediates relative to their mother compounds. The BOD_5_/COD ratio for untreated 100 mg L^−1^ 2,4-DCP was lower than that for 4-CP, implying that 2,4-DCP exhibited much higher toxicity relative to 4-CP. This is not surprising since the toxicity of chlorophenolic compounds was reported to increase with an increase in the number of Cl constituents on the aromatic structure (Kantar et al. 2019). However, following Fenton treatment using an optimum ZVI and pyrite dose, the BOD_5_/COD ratio increased from 0.1 to 0.82 for 2,4-DCP and from 0.15 to 0.66 for 4-CP, suggesting that the Fenton degradation significantly improved the biodegradability of both 4-CP and 2,4-DCP. Kayan et al. (2021) stated that the Fenton treatment using pyrite as the sole catalyst significantly improved the biodegradability of chlorophenolic compounds under aerobic conditions in the order of 2,4-DCP > 4-CP.

## Conclusions

Zero-valent iron has been reported to be a promising catalyst in the heterogeneous Fenton process. However, its efficacy decreases due to particle aggregation and surface passivation with surface oxidation products. Here, the influence of pyrite on chlorophenol (CP) degradation by the Fenton process with micron-sized ZVI as the catalyst was investigated in batch reactors. Our results indicate that while the CP treatment with ZVI as the sole catalyst in the Fenton process was adversely affected by rapid surface passivation and the aggregation of ZVI particles, the addition of pyrite to systems with ZVI particles significantly improved the oxidative treatment of both 4-CP and 2,4-DCP. The CP removal mainly occurred through the oxidative degradation of both 4-CP and 2,4-DCP with some strong radicals such as hydroxyl radicals (•OH) in solution and on the catalyst surface while the adsorption onto the catalyst surface was only responsible for 10 to 25% of CP removals, depending on the type of CP studied. The enhanced CP removal by the ZVI/H_2_O_2_ system in the presence of pyrite was attributed to the ability of pyrite to (1) create an acidic environment for optimum Fenton process, (2) provide support material for ZVI to minimize ZVI particle agglomeration, and (3) stimulate Fe redox cycling for enhanced surface site generation. Following Fenton treatment, the Fenton degradation by-products of 4-CP and 2,4-DCP became drastically more biodegradable relative to their mother compounds. Overall, despite the positive results obtained using synthetic wastewater containing CPs in the current study, the efficacy of this method must be confirmed using actual industrial wastewater under continuous flow conditions similar to those observed in the field.

### Supplementary Information

Below is the link to the electronic supplementary material.

Supplementary file1 (DOCX 1904 KB)

## Data Availability

All data will be available upon request from the corresponding author.
