# Supplementary material for: Effect of pyrite on the treatment of chlorophenolic compounds with zero-valent iron-Fenton process under uncontrolled pH conditions: reaction mechanism and biodegradability
Source: Environ Sci Pollut Res Int. 2024 Jul 15;31(35):47836–50. doi: 10.1007/s11356-024-34329-z (PMC11297809; doi:10.1007/s11356-024-34329-z)
Supplement: Supplementary file 1 — Supplementary file1 (DOCX 1904 KB) [file 11356_2024_34329_MOESM1_ESM.docx]

**Supporting Information**

**Effect of pyrite on the treatment of chlorophenolic compounds with zero valent iron-Fenton process under uncontrolled pH conditions: Reaction mechanism and biodegradability**

Ozlem Oral^1^, Cetin Kantar^1,*^ and Ilker Yildiz^2^

^1^Canakkale Onsekiz Mart University, Department of Environmental Engineering, 17100 Canakkale Turkey

^2^Middle East Technical University, Central Laboratory, 06800 Ankara Turkey

**Fig. S1** The XRD pattern of natural pyrite.


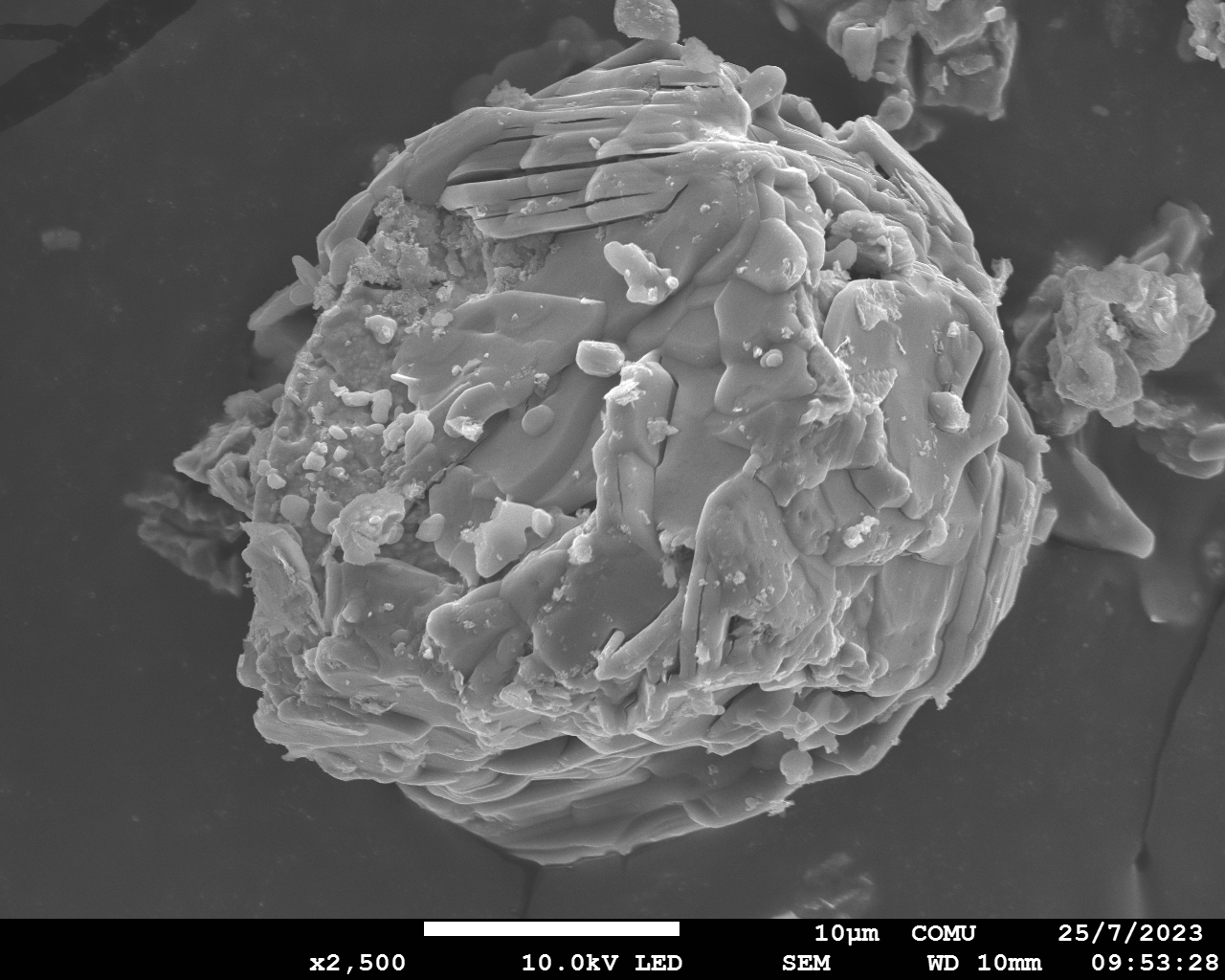

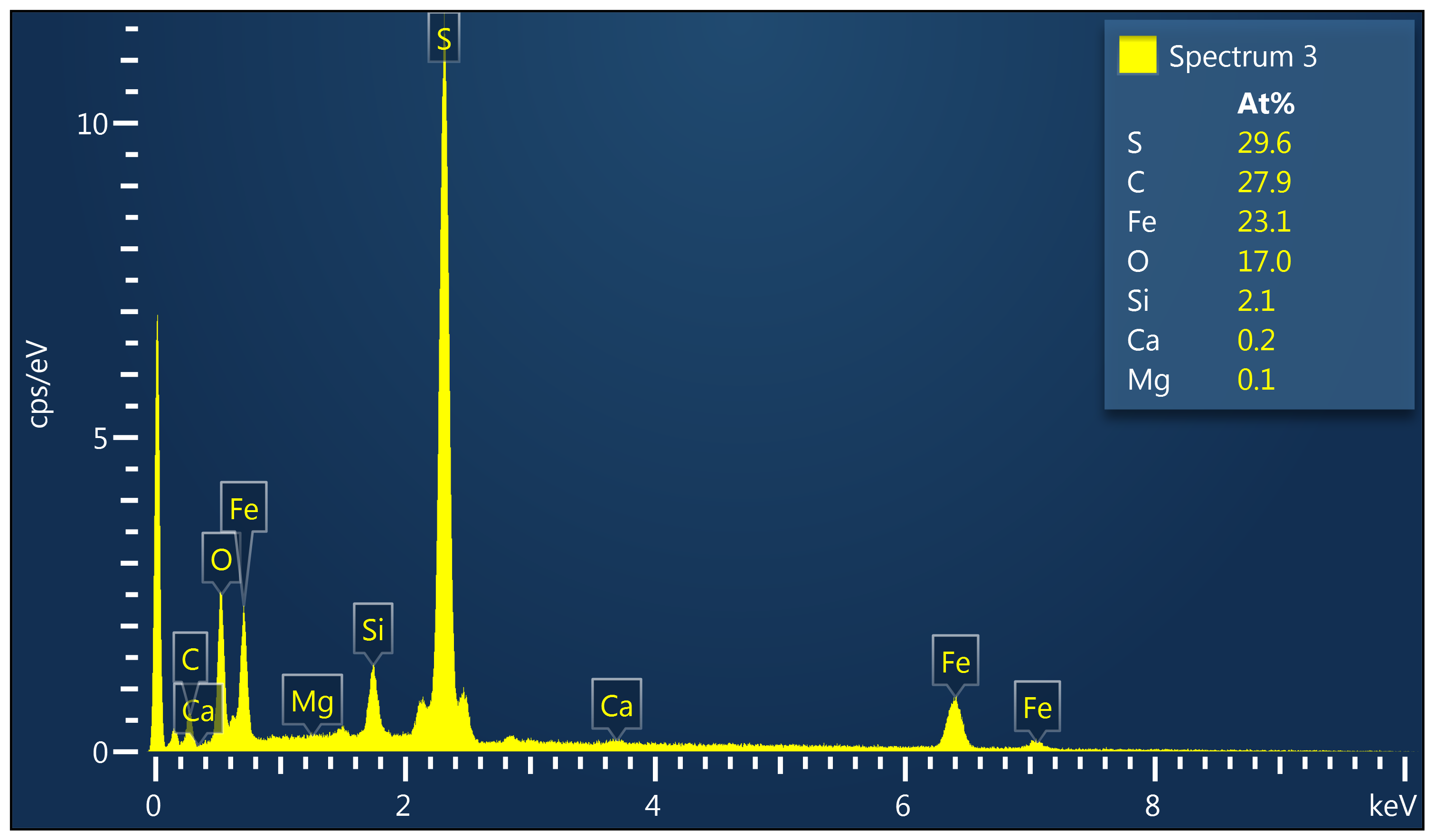


**(b)**

**(a)**

**Fig. S2** Raw pyrite SEM image (a) and EDS spectra (b).

**Fig. S3** XRD spectra of zero-valent iron particles.

**Fig. S4** The effect of hydrogen peroxide (H_2_O_2_) concentration on first order reaction kinetics for 2,4-DCP removal. The initial pH was 5.2. The experiment contained a pyrite dose of 0.8 g L^-1^ and a ZVI dose of 0.2 g L^-1^.

**Surface Characterization**

The solid samples taken from the batch reactors at desired reaction times were analyzed with X-Ray Photoelectron Spectroscopy (XPS) to determine oxidation products on pyrite/ZVI surface during ZVI-Fenton oxidation of 4-CP and 2,4-DCP in the presence of pyrite. Surface analysis was carried out in a PHI 5000 Versa Probe XPS (Φ ULVAC-PHI, Inc., Kanagawa, Japan) using a microfocused (100µm, 17.6W) monochromatized AlKa  radiation (1486.6 eV) as an X-ray anode. The pressure inside the main chamber was maintained at 10^-7^ Pa.  The binding energy scale was referenced by setting the C-H peak maximum in the C_1s_ spectrum to 284.6 eV, and the atomic composition was estimated using a Multipak software. High resolution spectra were peak fitted by using CASA XPS software. Shirley background was used prior to any peak-fitting procedure. A Gaussian–Lorentzian sum function in 70:30 ratios was used to fit the individual peak.

The solid samples taken from the batch degradation experiments before and after reaction were analyzed using scanning electron microscope (SEM) equipped with energy dispersive spectroscopy (EDS) (JEOL SEM-7100-EDX) to obtain information on surface morphology and elemental composition of pyrite and ZVI surface.

The solid samples withdrawn from batch degradation experiments were also analyzed with XRD (PANalytical Empyrean) to identify the purity of pyrite and ZVI used in the experiments, and confirm the form of surface oxidation species during ZVI-Fenton oxidation of 4-CP and 2,4-DCP in the presence of pyrite.

The solid samples withdrawn from batch degradation experiments were analyzed with FTIR (Thermo, Nicolet-iS10) over a scan range of 4000-650 cm^-1^ to determine the types of surface functional groups present on ZVI/pyrite surface before and after reaction.

The salt titration method, described by Oral et al. (2022), was used to determine the effects of reaction time on ZVI/pyrite surface charge during batch degradation experiments. In short, 30 mL ZVI/pyrite suspension was withdrawn from the reactors at desired reaction time intervals, and immediately loaded into 50 mL falcon tubes for salt titration experiments. Prior to performing the salt titration, the initial pH of the suspension was measured, and a desired amount of 1 M NaCl was added to the tubes to adjust the ionic strength to first 0.01 M, and then to 0.1 M, and allowed to come to equilibrium. The final pH values were measured after each ionic strength adjustment, and the results were plotted in terms of ∆pH (the differences between the final and initial pH values) *vs.* pyrite and ZVI mass doses or reaction times.

**Analytical Measurements**

Chlorophenols and their aromatic (e.g., organic acids, hydroquinone, benzoquinone) and aliphatic (e.g., acetic acid, formic acid) oxidation products were analyzed using HPLC (Shimadzu LC-20AT) equipped with SPD-M20A diode array detector and InterSustain C-18 column (4.6x150 mm). The operating conditions for HPLC analysis are provided in Table S1. The HPLC methods for the analysis of chlorophenolic compounds were established according to the manufacturer’s instructions of InterSustain C-18 column. The mobile phases were 50 % methanol/0.1 % H_3_PO_4_ (Line A) and 100 % methanol (Line B). The operating conditions for the gradient program were: 100 % Line A for 10 min. and a linear gradient evolution from 100 % Line A to 20 % Line A (80 % Line B) in 20 min. The flowrate was 1 mL min^-1^, and the injection volume was 40 µL. The column temperature was set to 30 ^o^C. The mobile phase used in the analysis of organic acids was 20 mM phosphate buffer adjusted to pH 2/acetonitrile (99:1, v/v). The measurement wavelength was 210 nm and the flow rate was 0.6 mL min^-1^ (Table S1). The HPLC operating conditions for aromatic compounds such as hydroquinone, benzoquinone and chlorohydroquinone can be found in Table S1.

The reaction products of 4-CP and 2,4-DCP oxidation with ZVI-Fenton process in the presence of pyrite were also analyzed with GC-MS using a procedure outlined by Kantar et al. (2019a). Prior to performing GC-MS measurements, the reaction intermediates were extracted from samples using n-hexane. The samples were first acidified to pH 2 with concentrated H_2_SO_4_, and extracted three times with 15 mL n-hexane. The extracted samples were then combined, and concentrated to 2 mL by evaporation at 40 ^o^C. The GC-MS analysis was performed on Shimadzu QP2010 Ultra equipped with an HP-5MS column (5 % phenyl methyl silox) 30 m x 0.25 mm x 0.25 µm capillary column using He as the carrier at a constant flow rate of 1 mL min^-1^. The oven temperature program was 6 min at 80 ^o^C , 4 ^o^C min^-1^ to 180 ^o^C, and 10 min at 180 ^o^C. The temperatures for the injection port and ion source remained constant at 280 and 230 ^o^C, respectively. The MSD scan range of 50 to 700 amu was selected, and the WILEY 7/NIST27/NIST147 library was used to determine the structural assignment of the identified compounds. In addition, analytical standard samples were also run to confirm the compounds as suggested by the library.

The concentration of chloride ion in aqueous samples was determined using spectrophotometric mercuric thiocyanate method (Hach Lange Method 20635-00). Hydrogen peroxide concentrations were measured with titanium sulfate method (Kantar et al. 2019).

The total iron contents of aqueous samples were determined using atomic absorption spectroscopy (PG Instruments AA500F), calibrated with 1000 mg L^-1^ AAS standard stock solutions. The Fe(II) contents of aqueous samples were determined by spectrophotometric 1.10-phenanthroline method (PG Instruments T90+) (Kantar et al. 2019).

**Table S1** Operating conditions for HPLC analysis.

| **Compounds** | **Retention time**  **(min)** | **Mobile phase** | **Flow,**  **mL/min** | **Column temp.** | **Injection Volume** | **Λ (nm)** | **Column** |
| --- | --- | --- | --- | --- | --- | --- | --- |
| 4-CP | 8.5 | A: %50 methanol:%50 water:%0.1 H_3_PO_4_  B: %100 methanol, Gradient flow,  0-10 min: A → B, % 100 A line; 10-20 min: A:B – 20:80; 20-40 min: A:B – 20:80. | 1 | 30°C | 10 µL | 280 | C18, 5µm,  4.6*150mm |
| 2,4-DCP | 15.9 |  |  |  |  |  |  |

| **Intermediate** | **Retention time (min)** | **Mobile phase** | **Flow,**  **mL/min** | **Column temp.**  **(^o^C)** | **Injection Volume** | **Λ (nm)** | **Column** |
| --- | --- | --- | --- | --- | --- | --- | --- |
| Hydroquinone | 3.7 | Water:methanol:acetic acid, 79.2:19.2:1, (v/v/v) | 0.8 | 40 | 40 µL | 280 | C18, 5µm,  4.6*150mm |
| Benzoquinone | 5.8 |  |  |  |  |  |  |
| Chlorohydroquinone | 7.9 |  |  |  |  |  |  |
| Formic Acid | 3.8 | 20 µM phosphate buffer (pH 2)/acetonitril, 99:1, (v/v) | 0.6 | 30 | 50 µL | 210 |  |
| Maleic Acid | 6.15 | Methanol/%0.1 phosphoric acid in water, 2/98, v/v | 1 | 30 | 20 µL | 214 | C18, 5µm,  4.6*250mm |
| Acetic Acid | 5.1 |  |  |  |  |  |  |

**Table S2** First order rate model parameters and model statistics at different ZVI and pyrite doses for 4-CP. The experimental data are given Fig. 1a.

| **Exp. #** | **4-CP (mg L^-1^)** | **Initial**  **pH** | **H_2_O_2_ (M)** | **ZVI (g L^-1^)** | **Pyrite**  **(g L^-1^)** | **k, min^-1^** | **R^2^** |
| --- | --- | --- | --- | --- | --- | --- | --- |
| **1** | 100 | 4.8 | 0.005 | 0 | 1 | 1.9316 | 0.98 |
| ***2*** | 100 | 4.8 | 0.005 | 0.2 | 0.8 | 2.0729 | 0.98 |
| **3** | 100 | 4.8 | 0.005 | 0.4 | 0.6 | 2.071 | 0.98 |
| **4** | 100 | 4.8 | 0.005 | 0.6 | 0.4 | 2.0179 | 0.98 |
| **5** | 100 | 4.8 | 0.005 | 0.8 | 0.2 | 0.8646 | 0.99 |
| **6** | 100 | 4.8 | 0.005 | 1 | 0 | 0.0197 | 0.99 |

**Table S3** First order rate model parameters and model statistics at different ZVI and pyrite doses for 2,4-DCP. The experimental data are given in Fig. 1b.

| **Exp. #** | **2,4-DCP (mg L^-1^)** | **Initial**  **pH** | **H_2_O_2_ (M)** | **ZVI (g L^-1^)** | **Pyrite**  **(g L^-1^)** | **k, min^-1^** | **R^2^** |
| --- | --- | --- | --- | --- | --- | --- | --- |
| **1** | 100 | 5.2 | 0.005 | 0 | 1 | 1.4987 | 0.95 |
| ***2*** | 100 | 5.2 | 0.005 | 0.2 | 0.8 | 1.6548 | 0.98 |
| **3** | 100 | 5.2 | 0.005 | 0.4 | 0.6 | 1.5622 | 0.98 |
| **4** | 100 | 5.2 | 0.005 | 0.6 | 0.4 | 0.9708 | 0.98 |
| **5** | 100 | 5.2 | 0.005 | 0.8 | 0.2 | 0.2325 | 0.98 |
| **6** | 100 | 5.2 | 0.005 | 1 | 0 | 0.0028 | 0.81 |

**Fig. S5** Comparison of CP removal from solution and Cl^-^ release from aromatic structure as a function of time in batch degradation experiments with: (a) 4-CP (0.2 g L^-1^ ZVI, 0.8 g L^-1^ pyrite, 100 mg L^-1^ 4-CP_,_ 0.005 M H_2_O_2_ and initial pH 4.8) and (b) 2,4-DCP (0.2 g L^-1^ ZVI, 0.8 g L^-1^ pyrite, 100 mg L^-1^ 2,4-DCP_,_ 0.005 M H_2_O_2_, and initial pH 5.2).

**(b)**

**(a)**

**H_2_O_2_**

**H_2_O_2_**

**H_2_O_2_**

**H_2_O_2_**

**Fig. S6** Comparison of chlorophenol removal in the absence or presence of hydrogen peroxide (H_2_O_2_) for: (a) 4-CP (0.2 g L^-1^ ZVI, 0.8 g L^-1^ pyrite and initial pH 4.8) and (b) 2,4-DCP (0.2 g L^-1^ ZVI, 0.8 g L^-1^ pyrite and initial pH 5.2). The initial 4-CP and 2,4-DCP concentration was 100 mg L^-1^ in all experiments.

**(B)**

**(a)**

**(b)**

**Fig. S7** The role of radical scavengers on 4-CP and 2,4-DCP removal in batch system containing: (a) 4-CP (0.2 g L^-1^ ZVI, 0.8 g L^-1^ pyrite, 0.02 M H_2_O_2_ and initial pH 4.8), and (b) 2,4-DCP (0.2 g L^-1^ ZVI, 0.8 g L^-1^ pyrite, 0.005 M H_2_O_2_ and initial pH 5.2). The initial 4-CP and 2,4-DCP concentration was 100 mg L^-1^ in all experiments.


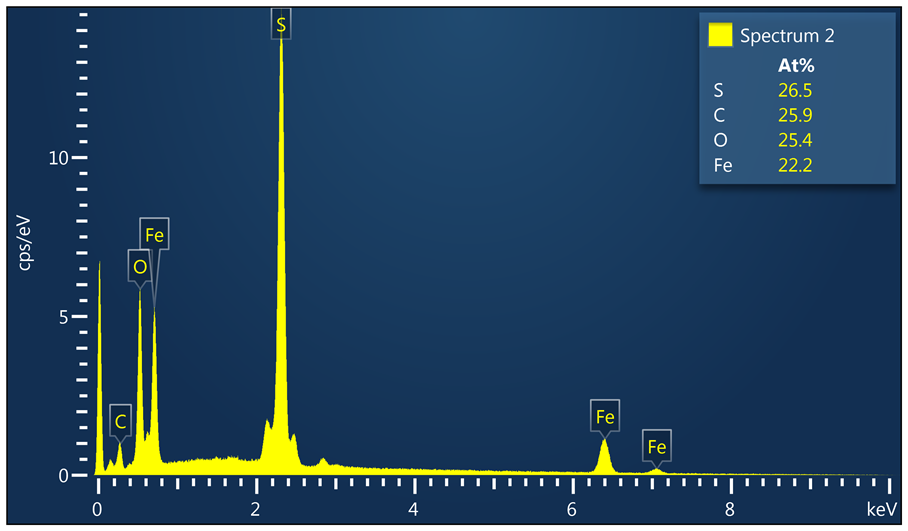

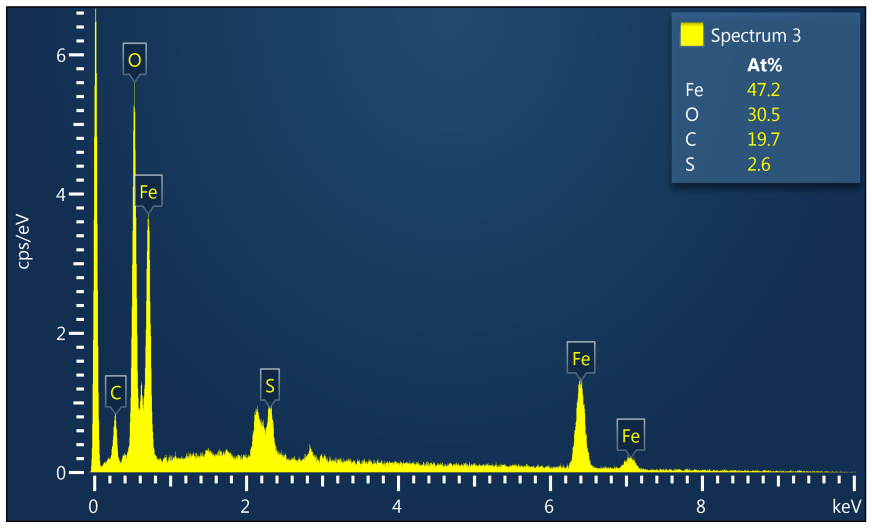

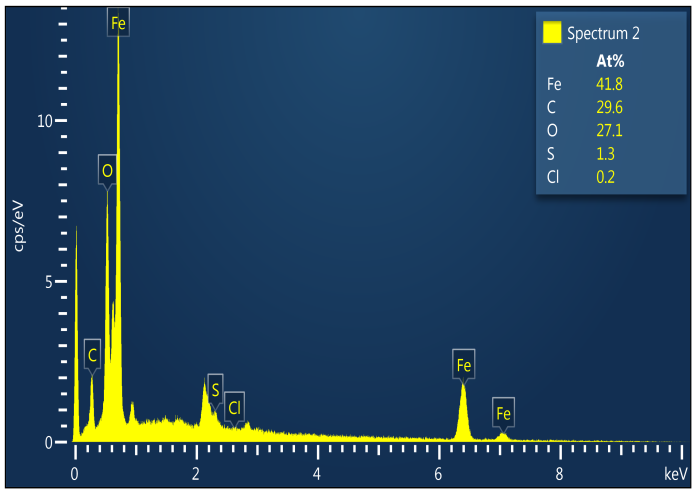


**(c)**

**(b)**

**(a)**

**Fig. S8** EDS spectra of solid samples taken from the batch reactor containing:. (a) 1 g L^-1^ pyrite + 0.005 M H_2_O_2_, (b) 0.2 g L^-1^ ZVI + 0.8 g L^-1^ pyrite + 0.005 M H_2_O_2_, and (c) 0.2 g L^-1^ ZVI + 0.8 g L^-1^ pyrite + 0.005 M H_2_O_2_ + 100 mg L^-1^ 2,4-DCP.

**Fig. S9** XRD spectra of pyrite and ZVI particles under different experimental conditions.

**Fig. S10** Comparison of total organic carbon (TOC) and CP removals in batch reactors containing: (a) 4-CP (0.2 g L^-1^ ZVI, 0.8 g L^-1^ pyrite, 0.005 M H_2_O_2_ and initial pH 4.8), and (b) 2,4-DCP (0.2 g L^-1^ ZVI, 0.8 g L^-1^ pyrite, 0.005 M H_2_O_2_ and initial pH 5.2). The initial 4-CP and 2,4-DCP concentration was 100 mg L^-1^ in all experiments.

**Fig. S11** Evolution of organic acids (a) and aromatic compounds (b) during Fenton degradation of 2,4-DCP in batch reactors containing 0.2 g L^-1^ ZVI, 0.8 g L^-1^ pyrite, 100 mg L^-1^ 2,4-DCP, 0.005 M H_2_O_2_ and initial pH 5.2.

**Fig. S12** Evolution of organic acids (a) and aromatic compounds (b) during Fenton degradation of 4-CP in batch reactors containing 0.2 g L^-1^ ZVI, 0.8 g L^-1^ pyrite, 100 mg L^-1^ 4-CP, 0.005 M H_2_O_2_ and initial pH 4.8.
